# Splenic Infarct with Polysplenia Syndrome

**DOI:** 10.5334/jbsr.2685

**Published:** 2022-06-24

**Authors:** Qin Ling Zhu, wenjun zhu

**Affiliations:** 1 The first hospital of jiaxing, CN

**Keywords:** polysplenia syndrome, isomerism, abdominal computed tomography, splenic infarction

## Abstract

A rare case of spontaneous splenic infarction with polysplenia is presented. The diagnosis was made by confirmed by enhanced computed tomography (CT), which showed multiple spleens in the left abdomen and one of the spleen showing low attenuation areas representing infarct. Polysplenia syndrome is a rare entity associated with heterotaxy syndromes. Radiological examinations help the diagnosis by identifying infarcts in the spleen and other abnormal organs in the chest and abdomen.

**Teaching Point:** We report a rare case of polysplenic syndrome with splenic infarction.

## Background

Polysplenic syndrome is a very rare syndrome consisting of congenital multiple system malformations, and a subtype of visceral ectopic syndrome. It is characterized by presence of two or more spleens and anomalies of other asymmetric organs. But splenic infarction rarely occurs.

Medical imaging advances have greatly enhanced our ability to detect and characterize these anomalies.

## Case History

A 72-year-old female patient was admitted in our hospital with a history of fever and abdominal pain. She denied previous episodes of similar pain presentation. She didn’t have any disease before.

**Figure 1:**
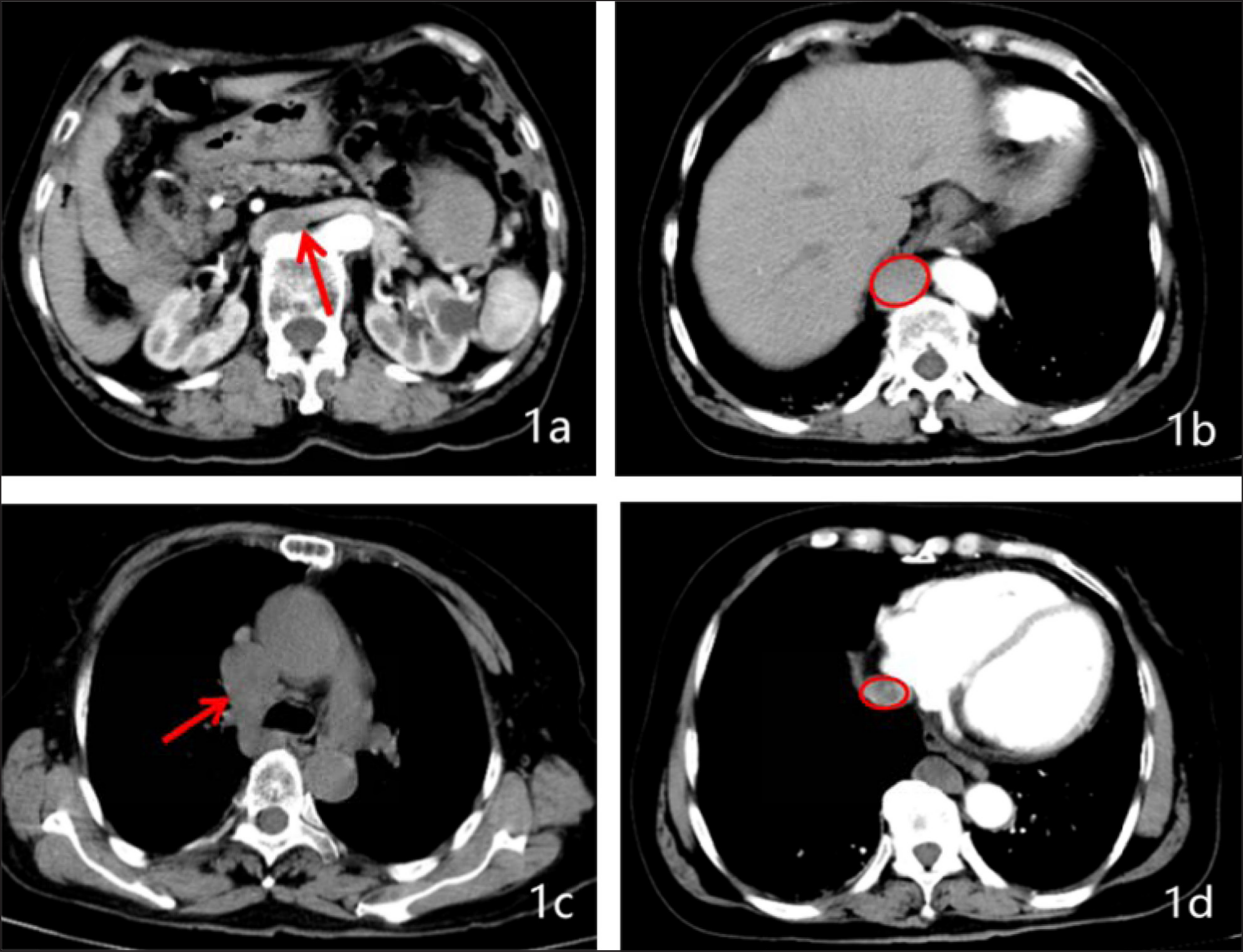
Drainage of the left renal vein into the IVC is shown **(1a)**. Inferior vena cava with azygos vein continuation on abdominal CT **(1b)**.

**Figure 2:**
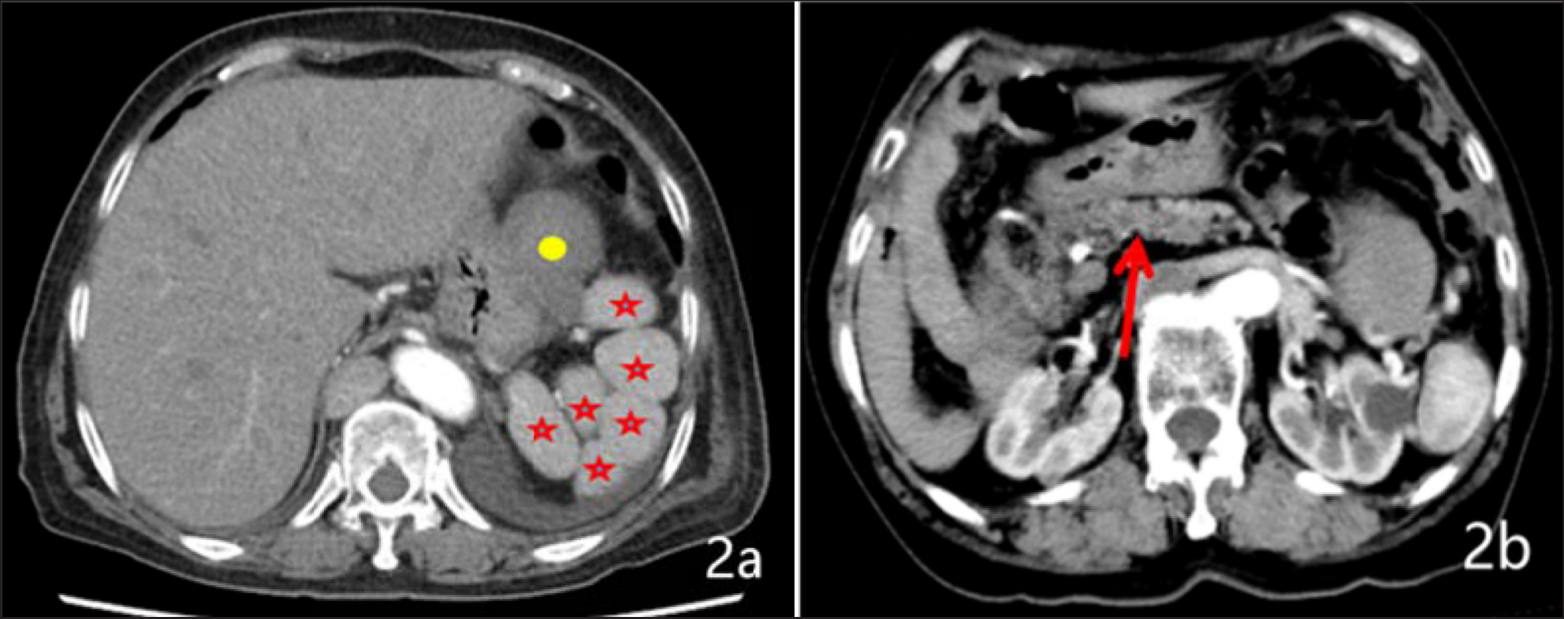
CT transverse section showing multiple spleens in the left upper quadrant along the greater curvature of the stomach (asterisks) **(2a)**, scan shows at a lower level the homogeneous hypodense mass (Round shape). The pancreas is truncated, with no pancreatic tail **(2b)**.

We performed a computed tomography (CT) scan for her and showed evidence of multiple spleens (6 in total) on the left side of the abdomen. There was also a low-density mass. The azygos vein was dilated and appeared to be the continuation of inferior vena cava (IVC) (***Figure 1b***) and drained into superior vena cava. The infrarenal segment of the IVC was normally positioned and after receiving the left and right renal vein continued as azygos vein passing into superior vena cava (***Figure 1a–b***). After draining the hepatic veins, the suprahepatic segment of IVC opened into right atrium (***Figure 1d***). The pancreas was short and truncated with the agenesis of part of the body and the tail (***Figure 2b***). Echocardiography showed dextrocardia with no other anomalies.

The diagnosis of polysplenia syndrome was made. In order to further confirm the diagnosis, enhanced CT was performed for the patient again. Contrast-enhanced CT confirmed the findings of polysplenia and non-enhancing hypodense areas in one spleen in the left lumber region, suggesting infarct.

**Figure 3:**
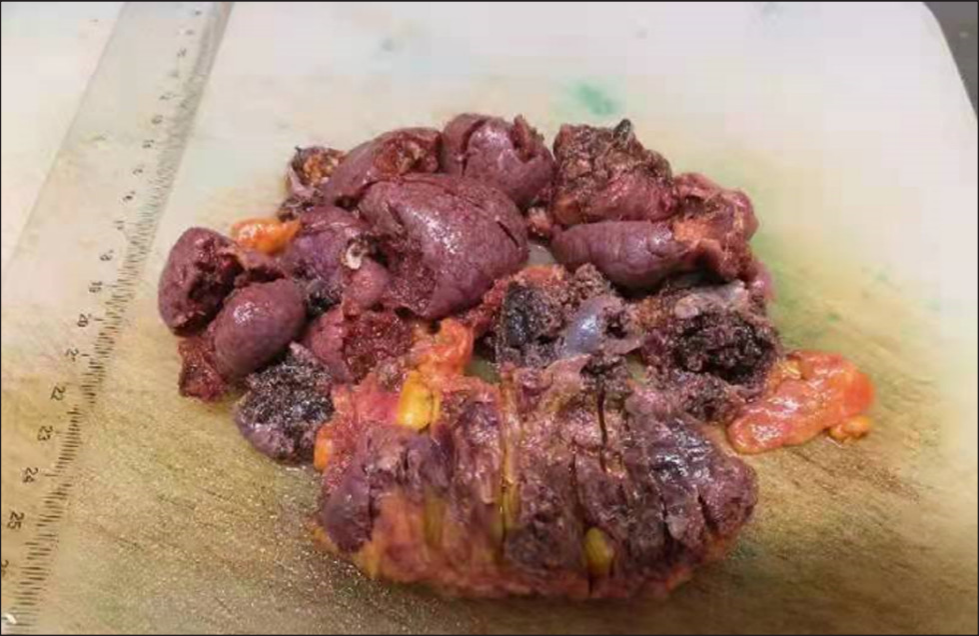
Laparoscopic surgery to remove multiple spleens.

The patient was operated on thereafter, and the spleen with infarct was removed. Laparoscopic surgery and many of bloody ascites were found intraoperatively. The enlarged spleen was dark red (***Figure 3***). Histopathology showed dilated and congested splenic sinusoids and changes of splenic infarction.

## Comment

Polysplenic syndrome (PS) is a rare congenital abnormality with an incidence of 1:250,000 to 6:250,000 live births in infants and young children [1]. Polyspleen can occur alone, but it is often associated with various malformations of the cardiovascular and internal organs. The incidence of adult PS patients cannot be estimated. It is more common in women. The severity of clinical symptoms and prognosis are mostly related to the severity of cardiovascular malformations. Most PS patients survive for less than five years, and a very small number of patients with normal or mild heart abnormalities can survive to adulthood.

We checked references to find that only a small number of cases of splenic torsion with polysplenia have been reported in the English-language literature to date. This is the only elderly patient.

Polysplenic syndrome visceral abnormalities are diversified; the cardiovascular system can be expressed as right heart, right aortic arch, isolated left ventricle, coarctation of the aorta, patent ductus arteriosus, abnormal right subclavian artery, interruption of the IVC with azygos continuation, anterior duodenal portal vein, ventricular septal defect, endocardial cushion defect, or sick sinus syndrome. The digestive system can appear as the right stomach, left liver, or intestinal malrotation. The pancreas is truncated, divided, circular, or poorly rotated, biliary atresia, spleen [2]. The respiratory system can be manifested as bilateral bilobal lungs (absence of horizontal fissures in the right lung). There may also be abnormalities of the urogenital system, manifested as horseshoe kidney, dysplasia or absent kidney, and abnormal ureter [3].

Early diagnosis and treatment can effectively avoid the occurrence of serious complications, such as sepsis, splenic gangrene, and so on [7], which is beneficial to the prognosis of patients.

Enhanced CT was a useful tool for preoperative diagnosis. The patient recovered well after splenectomy without complications.

When polysplenic syndrome patients have pain in the left upper abdomen with fever, beware of splenic infarction. Splenic infarction is difficult to diagnose in polysplenic syndrome patients, the disease progresses quickly, and the optimal treatment time for patients is easily delayed and life-threatening. It is very important to raise the awareness of radiologists on this disease.
